# Assessment of enzymatically synthesized DNA for gene assembly

**DOI:** 10.3389/fbioe.2023.1208784

**Published:** 2023-07-05

**Authors:** Brooke L. Simmons, Nathan D. McDonald, Natalie G. Robinett

**Affiliations:** ^1^ U.S. Army Combat Capabilities Development Command (DEVCOM) Chemical Biological Center, Gunpowder, MD, United States; ^2^ Oak Ridge Institute for Science and Education (ORISE), Oak Ridge, TN, United States; ^3^ Excet, Inc., Springfield, VA, United States

**Keywords:** enzymatic DNA synthesis, *de novo* DNA, gene assembly, chemical DNA synthesis, benchtop DNA synthesis

## Abstract

Phosphoramidite chemical DNA synthesis technology is utilized for creating *de novo* ssDNA building blocks and is widely used by commercial vendors. Recent advances in enzymatic DNA synthesis (EDS), including engineered enzymes and reversibly terminated nucleotides, bring EDS technology into competition with traditional chemical methods. In this short study, we evaluate oligos produced using a benchtop EDS instrument alongside chemically produced commercial oligonucleotides to assemble a synthetic gene encoding green fluorescent protein (GFP). While enzymatic synthesis produced lower concentrations of individual oligonucleotides, these were available in half the time of commercially produced oligonucleotides and were sufficient to assemble functional GFP sequences without producing hazardous organic chemical waste.

## Introduction

Phosphoramidite solid-phase chemical DNA synthesis (CDS) (Beaucage and Caruthers, 1981) is the standard method for producing *de novo* single-stranded DNA (ssDNA) and is used on a large scale by commercial vendors to produce oligonucleotides (oligos) for PCR, cloning, and increasingly for *de novo* construction of genes and plasmids (Kosuri and Church, 2014; Song et al., 2021). CDS methods have evolved to maximize product length while minimizing overall cost and errors, but they are limited to production of oligos of 200 or fewer nucleotides (nt) and require the use of hazardous chemicals such as acetonitrile, tetrahydrofuran, and dichloromethane (Hughes et al., 2011; Kosuri and Church, 2014; Song et al., 2021).

Controlled enzymatic DNA synthesis (EDS) was first developed in the 1950s–1970s (Grunberg-Manago et al., 1955; Bollum, 1962; Chang and Bollum, 1971) with the discovery of the terminal deoxynucleotidyl transferase (TdT) enzyme that adds dNTPs to the 3′-hydroxyl group of DNA. Recent breakthroughs have addressed historical limitations of TdT (Palluk et al., 2018; Song et al., 2021) by evolving more thermally stable TdT enzymes (Chua et al., 2020; Lu et al., 2022), developing reversible 3′-dNTP protecting groups that can be accommodated by TdT (Mathews et al., 2016; Mathews et al., 2017; Hoff et al., 2020), and improving enzymatic coupling efficiency to enable a cyclic, solid-phase-templated extension of DNA (Palluk et al., 2018; Barthel et al., 2020; Hoff et al., 2020). EDS has the added benefit of eliminating the use of hazardous organic chemicals by utilizing a primarily aqueous workflow (Song et al., 2021).

Several EDS-focused companies were launched within the last decade, including Molecular Assemblies and Nuclera (2013), DNA Script (2014), Camena Bioscience (2016), Ansa Biotechnologies (2018), and Kern Systems (2019) (Eisenstein, 2020). Camena Bioscience and DNA Script report greater than 99% coupling efficiency while producing 300- and 280-mer oligos, respectively (Eisenstein, 2020), elevating this technology to a competitive level with chemical synthesis (Masaki et al., 2022). More recently, Ansa Biotechnologies, Inc., reported the enzymatic synthesis of the longest *de novo* oligonucleotide ever produced in a single synthesis at 1,005 nucleotides long (Ansa Biotechnologies, 2023), and Camena Bioscience reported the production of a whole 2.7-kb plasmid using multi-enzymatic *de novo* DNA synthesis and gene assembly (Camenabio, 2020). However, EDS products are not readily available yet for purchase from commercial sources, and interested users are limited to early access programs (Ansa and Molecular Assemblies) or purchasing a benchtop EDS instrument (DNA Script).

The Syntax-100™ from DNA Script is the first commercially available benchtop EDS instrument. It is marketed to have a user-friendly platform and essentially aqueous waste stream and can synthesize 96 × 60 mers in 13 hours, including cleanup and quantitation. Here, we evaluate the Syntax-100™ to produce oligos using EDS and compare them to commercial CDS oligos to assemble a synthetic gene encoding green fluorescent protein (GFP). In this brief study, we consider the time of oligo synthesis and gene assembly, cost per oligo/base, sequence accuracy, and waste production.

## Materials and methods

### Oligo design and production

Primerize (Tian and Das, 2017) was used to design overlapping oligos for a GFP-coding sequence (Snapgene, 2023) with 20 nt overlap, 60 or 90 nt maximum length, and a minimum annealing temperature of 60°C. A set of twelve (90 nt, 100 nmol) or twenty (60 nt, 25 nmol) commercial CDS oligos was purchased from Integrated DNA Technologies (IDT, USA). Each set of CDS oligos was used for independent assembly experiments. The Syntax-100™ enzymatic DNA synthesizer was set up according to the user instructions, with reagents and consumables provided for a 60-mer kit. A set of twenty oligos (60 nt max) and Gibson assembly primers were synthesized in two or more replicates per plate on the Syntax-100™ at 0.3 nmol per oligo, normalized to a final concentration of 5 μM. After completion of each run (13 hours), the oligos were immediately stored at −20°C. Two individual plates of EDS oligos were produced and used for separate assembly experiments. Supplementary Table S1 lists all oligos used in this study.

### Gene assembly

Method modified from Dormitzer et al. (2013). A set of twelve (90 nt) CDS oligos, twenty (60 nt) CDS oligos, or twenty (60 nt) EDS oligos was pooled by combining 2 µL of each 5 µM oligo. A volume of 10 μL of ×2 Gibson Assembly Master Mix (New England Biosciences, #E2611) was combined with 10 µL of pooled oligos and incubated at 50°C/30 min. Next, 5 µL of this mixture was added to 12.5 µL of ×2 Q5 Master Mix (NEB, # M0492S), 2.5 µL each end oligo, and H_2_O to reach a final concentration of 25 µL. The PCR cycles were run as follows: 98°C/60 s, 98°C/10 s, 60°C/30 s, and 72°C/1.5 min, return to step 2 for 24 cycles, followed by 72°C/5 min and 10°C hold. An enrichment PCR using 2 µL of PCR1 and 2.5 µL of 5 µM end oligos was performed under the aforementioned conditions to obtain a full-length *gfp* product.

### TOPO cloning, transformation, colony screening, and sequencing

Following gel confirmation of the assembled gene product, 1 µL of Taq polymerase (NEB, #M0273S) was added to PCR to add a single deoxyadenosine (A) to the 3′-ends. TOPO cloning and transformation were performed using a TOPO^®^ TA Cloning^®^ Kit (Invitrogen #K457540) (Thermo Fisher Scientific, 2023). Transformants were screened by PCR, and plasmids were isolated via a Miniprep Kit (Zymo, #D4211) and were sequenced (Eurofins).

### Gibson assembly and GFP expression/fluorescence analysis

Confirmed, correct *gfp* sequences from each assembly were cloned into an expression vector using Gibson assembly primers (Supplementary Table S1). Separate PCRs were run with 2 μL of the template (pET28a (+) or PCR4_GFP), 25 μL of ×2 Q5 Master Mix, 2.5 μL of each 5 μM forward or reverse primer, and H_2_O to 50 μL using the online Q5 PCR protocol (New England Biolabs, 2023). Vector and insert sequences were gel-extracted and cloned in a Gibson assembly reaction according to the online protocol (New England Biolabs, 2023) and transformed into NEB5α competent cells (NEB, #C2987). Sequence-confirmed plasmids were transformed into BL21 DE3 competent cells (Thermo Fisher Scientific, #EC0114) and plated on LB/kanamycin plates. Cells transformed with pET28-GFP from each assembly were sub-cultured onto fresh LB/kanamycin plates with or without the addition of 10 mM isopropyl β-d-1-thiogalactopyranoside (IPTG), incubated overnight at 37°C, and imaged under UV light to confirm the expression of fluorescent GFP.

## Results and discussion

In this study, we sought to compare CDS- and EDS-sourced oligonucleotides for use in the assembly of the gene encoding GFP. We used the application Primerize (Tian and Das, 2017) to divide a GFP coding sequence (Snapgene, 2023) into overlapping oligos of 60 nt or 90 nt maximum length, a 20 nt overlap, and a minimum annealing temperature of 60°C that were amenable to assembly using previously established polymerase cycling assembly (PCA) methods (Supplementary Table S1) (Stemmer et al., 1995; Dormitzer et al., 2013). The 60 nt oligos provided the maximum length available from our EDS benchtop instrument, and the 90 nt oligos provided a longer yet cost-effective option for CDS oligos that could be used to compare the effects of oligo length on gene assembly. We synthesized replicates of twenty 60 nt oligos via EDS in two independent runs on the Syntax-100™. The total run time for each plate was thirteen hours, including sample cleanup and quantitation. A set of twenty (60 nt max, 25 nmol each) and twelve (90 nt max, 100 nmol each) commercial CDS oligos was ordered and delivered in 2 days.

**FIGURE 1:**
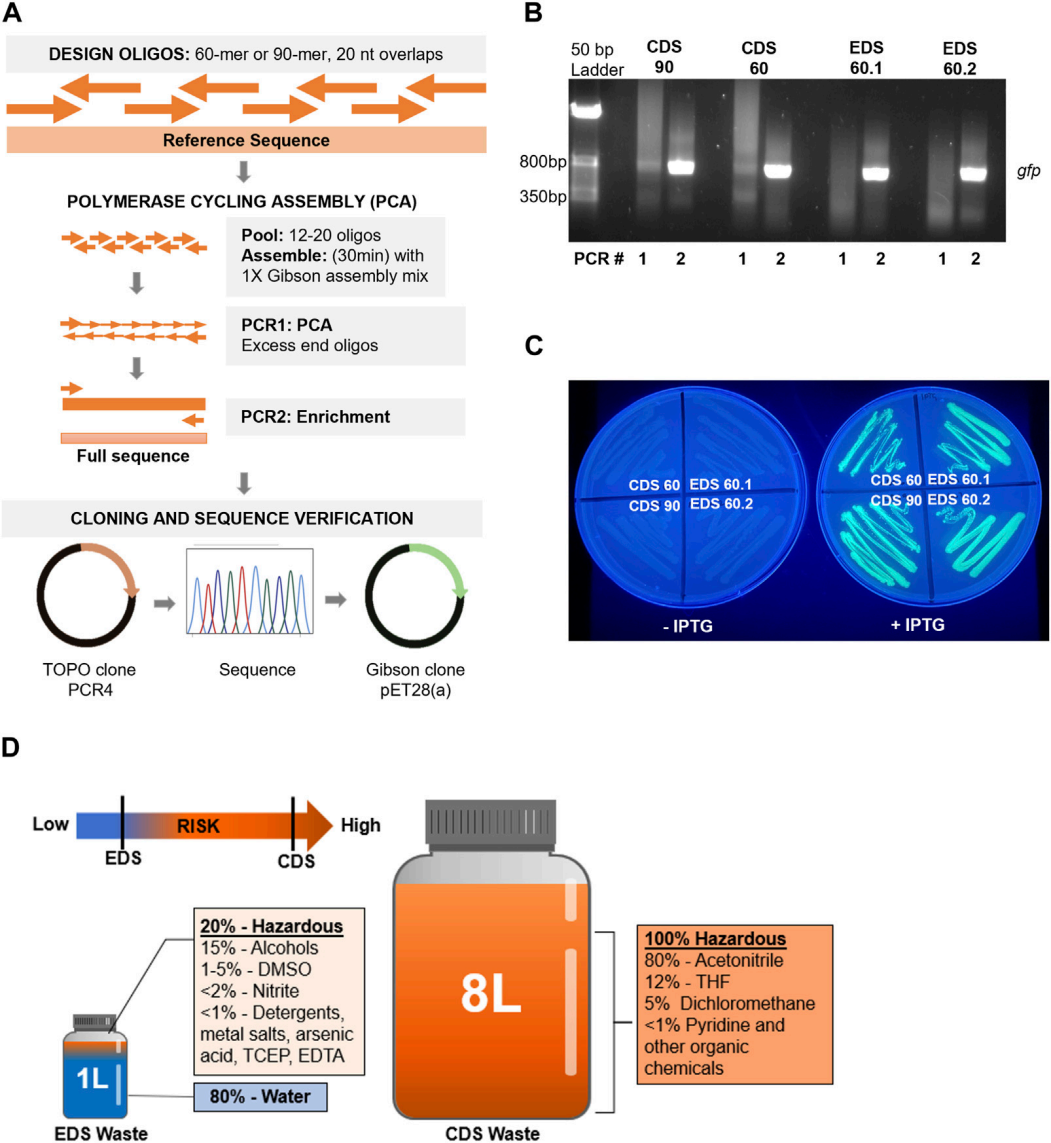
**(A)** Workflow for oligo design, gene assembly, cloning, sequence verification, and sub-cloning of synthetic *gfp* sequences, created with BioRender.com. **(B)** Pooled EDS (60 nt) and CDS (90 nt or 60 nt) oligos were assembled into *gfp* using an isothermal incubation step, followed by PCR1 for polymerase cycling assembly and PCR2 to enrich the final product (717 bp). **(C)** Sequence-verified GFP-encoding sequences assembled from each oligo set were cloned into a pET28 vector and transformed into BL21 DE3 *E. coli* that were grown on LB/kanamycin plates in the presence or absence of 10 mM IPTG; plates were imaged under UV light to demonstrate GFP expression in induced samples (right) compared to uninduced samples (left). **(D)** Analysis of waste stream for a benchtop EDS instrument (Syntax-100™) and CDS instrument (MerMade 192e) with the hazardous nature of waste indicated by color (non-hazardous = blue; hazardous = orange). Note: components and volumes are from production of one set of 96 × 60-mers per instrument.

Our adapted gene assembly approach (Dormitzer et al., 2013) included an initial isothermal assembly of pooled oligos, followed by two rounds of PCR: PCR1 for PCA and PCR2 to enrich the final product (Figure 1A). The CDS 90 and 60 nt oligo assemblies showed a faint band at the expected size (717 bp) after PCR1 and a clear band after PCR2, whereas the two EDS 60 nt oligo sets did not produce a visible full-length product until PCR2 (Figure 1B). Full gene products assembled from each oligo set were TOPO-cloned into pCR4 vectors (Thermo Fisher Scientific, 2023) and sequenced after transformation and colony screening. A minimum of ten transformants were screened and sequenced for each independent assembly (Table 1). We observed that the designed *gfp* sequence was successfully assembled using each of the four oligo sets (Table 1). Notably, the longer 90 nt CDS oligos did produce a higher percentage of correct sequences (40%) compared to the 60 nt oligo sets (27%, CDS and 24%, EDS). Sequence-validated *gfp* gene products from each oligo set were sub-cloned into an expression vector, transformed into competent *E. coli* cells, and analyzed for GFP protein expression and fluorescence. EDS- and CDS-sourced DNA sequences successfully produced functional gene products with confirmed expression and fluorescence of GFP (Figure 1C).

**TABLE 1:** Summary of gene assembly and sequence results from enzymatically or chemically synthesized oligos (EDS or CDS) and comparison with published results from the vendor.

	Gene assembly and sequencing results			Vendor results (DNAScript, 2022)	
Oligo source	EDS	CDS	CDS	EDS	CDS
Protein coding ORF	GFP	GFP	GFP	eGFP	eGFP
Oligo length (nt)	**51–60**	**51–60**	**47–90**	**59–60**	**59–60**
Overlap (nt)	20	20	20	nd	nd
Polymerase	Q5	Q5	Q5	Q5	Q5
Assembly method	One-step PCA	One-step PCA	One-step PCA	Two-step PCA	Two-step PCA
Error correction	No	No	No	Yes	Yes
Set of oligos produced	2	1	1	4	4
Independent assemblies	2	1	1	5	5
Transformants screened	44	17	15	480	480
Total sequenced	30 (70%)	15 (88%)	10 (67%)	∼384 (80%)	∼384 (80%)
# Correct sequences	7	4	4	nd	nd
% correct sequences	**23%**	**27%**	**40%**	**24%**	**18%**
1-bp SNPs or INDELS	13	4	2	nd	nd
Multiple SNPs or INDELS	10	7	4	nd	nd

Bolded values are for emphasis of oligo length and associated percentage of correctly assembled sequences.

While this work was being completed, the vendor for the Syntax-100™ published an application note on their website detailing a similar set of experiments (DNA Script, 2022). We have summarized those findings in Table 1 along with our independent validation of EDS oligos compared to commercially prepared CDS oligos. Using an analogous gene assembly method, we obtained similar percentages of correctly assembled sequences using EDS oligos (23%) compared to the vendor (24%). However, our results for CDS 60 nt oligos differed, with 27% of our sequenced transformants having the designed sequence compared to only 18% reported by the vendor. The vendor does not state the source of their CDS oligos.

Currently, the most cost-effective way to utilize the Syntax-100™ is to produce 96 × 60-mers (or 80-mers and 120-mers that have recently become available), which is comparable to ordering the equivalent number, length, and quality of oligos from IDT (Table 2) at approximately $0.20–$0.40 per base, if labor and the initial cost of the instrument are not included. The yield for EDS oligos is much lower (0.3 nmol per oligo per well), but in our experiments, EDS oligos were of sufficient quality and quantity to assemble a functional *gfp* gene. It is worth noting that purchasing a CDS synthetic *gfp* gene from commercial sources currently costs approximately $280, which is still substantially cheaper than purchasing or producing the assembly oligos by EDS or CDS methods. Finally, we were interested in analyzing the waste generated in our laboratory on a benchtop CDS instrument (MerMade 192E™, LGC Limited) compared to the Syntax-100™ after production of 96 × 60-mers. Our analysis indicated a clear reduction in hazardous chemical waste production in both volume and individual waste components when using the benchtop EDS method (Figure 1D).

**TABLE 2:** Analysis of cost, time, and accuracy of GFP sequences assembled from oligos produced via enzymatic (EDS) or chemical (CDS) DNA synthesis.

Oligo source	CDS			EDS				
Synthesis/delivery time	2 days			13 hours				
Oligo # produced	12	24	96	12	24	96	96	96
Oligo yield (nmol)	100	25	25	0.3	0.3	0.3	0.3	0.3
Oligo length (max, nt)	90	60	60	60	60	60	80	120
Cost/kit or order	$772	$540	$2,159	$2,470	$2,470	$2,470	$1,600	$2,550
Avg. cost per oligo	$64	$22	$22	$206	$103	$26	$16	$27
Avg. cost per base	$0.71	$0.37	$0.37	$3.43	$1.72	$0.43	$0.20	$0.22
Assembly time	1–2 days	1–2 days				1–2 days		
% perfect sequences	40% (n = 10)	27% (n = 15)				23% (n = 30)		

This brief study provides an independent validation that EDS oligonucleotides are suitable for use in gene synthesis workflows, with 23% of assembled samples containing the designed *gfp* sequence (Table 1). Our findings are similar to those described by the vendor in their recently published application note (DNA Script, 2022). Notably, we obtained similar results without adding a correction step in our PCA protocol. Our results for CDS gene assembly indicate a higher rate of correct sequences assembled (40% and 27% compared to 23% for EDS oligos) that may be due to longer starting oligo length (in the case of CDS 90 nt oligos) or the higher reported error rate for EDS (0.4%) *versus* CDS (0.1%) (Masaki et al., 2022). Further experiments are required to differentiate between errors introduced during DNA synthesis or gene assembly. Both EDS- and CDS-produced oligos used in gene assembly resulted in functional GFP-coding sequences.

Although commercial CDS sources remain the most cost-effective solution for obtaining oligos, it is worth noting that benchtop DNA synthesis instruments (CDS and EDS) provide users with improved access to on-demand DNA synthesis in mobile or austere laboratory settings. In our studies, EDS produced substantially less hazardous waste than a CDS instrument, suggesting this technology could provide a safer and more environmentally friendly alternative for large-scale DNA synthesis. Finally, the recent report of kilobase-length DNA by EDS methods (Ansa Biotechnologies, 2023) suggests that this technology may overcome the inherent length limitations faced by CDS methods. Future studies may include testing additional EDS kits, offering oligo modifications such as the addition of biotin, fluorophores, and quenchers for a variety of downstream applications.

## Data Availability

The original contributions presented in the study are included in the article/Supplementary Material; further inquiries can be directed to the corresponding author.

## Abbreviations

CDS, chemical DNA synthesis; EDS, enzymatic DNA synthesis; NT, nucleotide; TdT, terminal nucleotidyl transferase; dNTPs, dinucleotide triphosphates; IDT, Integrated DNA Technologies; DNA, deoxyribonucleic acid; GFP, green fluorescence protein; SNP, single-nucleotide polymorphism; INDEL, insertion/deletion; IPTG, isopropyl β- d-1-thiogalactopyranoside.
