# Supplementary material for: Assessment of enzymatically synthesized DNA for gene assembly
Source: Front Bioeng Biotechnol. 2023 Jul 5;11:1208784. doi: 10.3389/fbioe.2023.1208784 (PMC10354541; doi:10.3389/fbioe.2023.1208784)
Supplement: Supplementary file 1 [file Table1.DOCX]

Supplementary Material

Assessment of Enzymatically Synthesized DNA for Gene Assembly

Brooke L. Simmons, Nathan D. McDonald, Natalie G. Robinett^*^

*** Correspondence: Natalie G. Robinett** natalie.g.robinett.ctr@army.mil

| **Supplementary Table 1. Oligonucleotide sequences designed using Primerize (1)** | |
| --- | --- |
| **Oligo design 1 (60 nt max, CDS and EDS)** | |
| GFP1 | ATGAGTAAAGGAGAAGAACTTTTCACTGGAGTTGTCCCAATTCTTGTTGAAT |
| GFP2 | TGACAGAAAATTTGTGCCCATTAACATCACCATCTAATTCAACAAGAATTGGGACAACTC |
| GFP3 | TGGGCACAAATTTTCTGTCAGTGGAGAGGGTGAAGGTGATGCAACATACGGAAAACTTAC |
| GFP4 | AGGTAGTTTTCCAGTAGTGCAAATAAATTTAAGGGTAAGTTTTCCGTATGTTGCATC |
| GFP5 | TGCACTACTGGAAAACTACCTGTTCCATGGCCAACACTTGTCACTACTTTCTCTTATGG |
| GFP6 | TCATATGATCTGGGTATCTTGAAAAGCATTGAACACCATAAGAGAAAGTAGTGACAAGTG |
| GFP7 | CTTTTCAAGATACCCAGATCATATGAAACGGCATGACTTTTTCAAGAGTGCCATGCCCGA |
| GFP8 | TCCCGTCATCTTTGAAAAATATAGTTCTTTCCTGTACATAACCTTCGGGCATGGCACTC |
| GFP9 | ACTATATTTTTCAAAGATGACGGGAACTACAAGACACGTGCTGAAGTCAAGTT |
| GFP10 | ACCTTTTAACTCGATTCTATTAACAAGGGTATCACCTTCAAACTTGACTTCAGCACGTGT |
| GFP11 | CCTTGTTAATAGAATCGAGTTAAAAGGTATTGATTTTAAAGAAGATGGAAACATTCTTGG |
| GFP12 | GTGAGTTATAGTTGTATTCCAATTTGTGTCCAAGAATGTTTCCATCTTCTTTAAAATC |
| GFP13 | CACAAATTGGAATACAACTATAACTCACACAATGTATACATCATGGCAGACAAACAAAAG |
| GFP14 | GTCTAATTTTGAAGTTAACTTTGATTCCATTCTTTTGTTTGTCTGCCATGATGTA |
| GFP15 | TGGAATCAAAGTTAACTTCAAAATTAGACACAACATTGAAGATGGAAGCGTTCAACTAGC |
| GFP16 | AGGGCCATCGCCAATTGGAGTATTTTGTTGATAATGGTCTGCTAGTTGAACGCTTCCA |
| GFP17 | TGGCGATGGCCCTGTCCTTTTACCAGACAACCATTACCTGTCCACACAATC |
| GFP18 | GGTCTCTCTTTTCGTTGGGATCTTTCGAAAGGGCAGATTGTGTGGACAGGTAATGGTT |
| GFP19 | CCCAACGAAAAGAGAGACCACATGGTCCTTCTTGAGTTTGTAACAGCTGCTGGGATTA |
| GFP20 | CTATTTGTATAGTTCATCCATGCCATGTGTAATCCCAGCAGCTGTTACAAACTCAA |
| pET28_fwd | caaatagtagGCTAGCATGACTGGTGGACAGCAAATGGG |
| pET28_rev | ctttactcatATGGCTGCCGCGCGGCAC |
| GFP_fwd | cggcagccatATGAGTAAAGGAGAAGAAC |
| GFP_rev | tcatgctagcCTACTATTTGTATAGTTCATCCATG |
| **Oligo design 2 (90 nt max, CDS only)** | |
| GFP1 | ATGAGTAAAGGAGAAGAACTTTTCACTGGAGTTGTCCCAATTCTTGTTGAATTAGATGGTGATGTTAATGGGCACA |
| GFP2 | ACCCTCTCCACTGACAGAAAATTTGTGCCCATTAACATCACCATCTA |
| GFP3 | CTGTCAGTGGAGAGGGTGAAGGTGATGCAACATACGGAAAACTTACCCTTAAATTTATTTGC |
| GFP4 | ACACCATAAGAGAAAGTAGTGACAAGTGTTGGCCATGGAACAGGTAGTTTTCCAGTAGTGCAAATAAATTTAAGGGTAAGTTTTCCGTA |
| GFP5 | TGTCACTACTTTCTCTTATGGTGTTCAATGCTTTTCAAGATACCCAGATCATATGAAACGGCATGACTTTTTCAAGAGTGCCATGCCCGA |
| GFP6 | CAAACTTGACTTCAGCACGTGTCTTGTAGTTCCCGTCATCTTTGAAAAATATAGTTCTTTCCTGTACATAACCTTCGGGCATGGCACTC |
| GFP7 | ACGTGCTGAAGTCAAGTTTGAAGGTGATACCCTTGTTAATAGAATCGAGTTAAAAGGTATTGATTTTAAAGAAGATGGAAACATTCTTGG |
| GFP8 | TTTGTTTGTCTGCCATGATGTATACATTGTGTGAGTTATAGTTGTATTCCAATTTGTGTCCAAGAATGTTTCCATCTTCTTTAAAATC |
| GFP9 | ACATCATGGCAGACAAACAAAAGAATGGAATCAAAGTTAACTTCAAAATTAGACACAACATTGAAGATGGAAGCGT |
| GFP10 | GGTAAAAGGACAGGGCCATCGCCAATTGGAGTATTTTGTTGATAATGGTCTGCTAGTTGAACGCTTCCATCTTCAATGTTGTGTCTA |
| GFP11 | TGGCCCTGTCCTTTTACCAGACAACCATTACCTGTCCACACAATCTGCCCTTTCGAAAGATCCCAACGAAAAGAGAGACCACATGGTCC |
| GFP12 | CTATTTGTATAGTTCATCCATGCCATGTGTAATCCCAGCAGCTGTTACAAACTCAAGAAGGACCATGTGGTCTCTCTTTTCGT |

**Reference**

1. Tian S, Das R. Primerize-2D: automated primer design for RNA multidimensional chemical mapping. Bioinformatics. 2017;33(9):1405-6.
